# The MOVECLIM – AZORES project: Bryophytes from Pico Island along an elevation gradient

**DOI:** 10.3897/BDJ.12.e117890

**Published:** 2024-02-08

**Authors:** Rosalina Gabriel, Leila N. Morgado, Paulo A. V. Borges, Márcia C. M. Coelho, Silvia C. Aranda, Débora S. G. Henriques, Cecília Sérgio, Helena Hespanhol, Fernando Pereira, Manuela Sim-Sim, Claudine Ah-Peng

**Affiliations:** 1 cE3c- Centre for Ecology, Evolution and Environmental Changes/Azorean Biodiversity Group, CHANGE – Global Change and Sustainability Institute, School of Agricultural and Environmental Sciences, University of the Azores, Rua Capitão João d´Ávila, Pico da Urze, 9700-042, Angra do Heroísmo, Azores, Portugal cE3c- Centre for Ecology, Evolution and Environmental Changes/Azorean Biodiversity Group, CHANGE – Global Change and Sustainability Institute, School of Agricultural and Environmental Sciences, University of the Azores, Rua Capitão João d´Ávila, Pico da Urze, 9700-042 Angra do Heroísmo, Azores Portugal; 2 IITAA - Instituto de Investigação e Tecnologias Agrárias e do Ambiente, Faculdade de Ciências Agrárias e do Ambiente, Universidade dos Açores. Capitão João d‘Ávila street, 9700-042, Angra do Heroísmo, Portugal IITAA - Instituto de Investigação e Tecnologias Agrárias e do Ambiente, Faculdade de Ciências Agrárias e do Ambiente, Universidade dos Açores. Capitão João d‘Ávila street, 9700-042 Angra do Heroísmo Portugal; 3 IUCN SSC Atlantic Islands Invertebrate Specialist Group, 9700-042, Angra do Heroísmo, Azores, Portugal IUCN SSC Atlantic Islands Invertebrate Specialist Group, 9700-042 Angra do Heroísmo, Azores Portugal; 4 IUCN SSC Species Monitoring Specialist Group, 9700-042, Angra do Heroísmo, Azores, Portugal IUCN SSC Species Monitoring Specialist Group, 9700-042 Angra do Heroísmo, Azores Portugal; 5 Museo Nacional de Ciencias Naturales, Madrid, Spain Museo Nacional de Ciencias Naturales Madrid Spain; 6 Banco Genético Vegetal Autóctone, Empresa Municipal Cascais Ambiente, Lisboa, Portugal Banco Genético Vegetal Autóctone, Empresa Municipal Cascais Ambiente Lisboa Portugal; 7 cE3c- Centre for Ecology, Evolution and Environmental Changes / Natural History & Systematics (NHS) CHANGE – Global Change and Sustainability Institute, Lisbon, Portugal cE3c- Centre for Ecology, Evolution and Environmental Changes / Natural History & Systematics (NHS) CHANGE – Global Change and Sustainability Institute Lisbon Portugal; 8 CIBIO, Centro de Investigação em Biodiversidade e Recursos Genéticos, InBIO Laboratório Associado / BIOPOLIS, Program in Genomics, Biodiversity and Land Planning, Campus de Vairão, Universidade do Porto, 4485-661, Vairão, Porto, Portugal CIBIO, Centro de Investigação em Biodiversidade e Recursos Genéticos, InBIO Laboratório Associado / BIOPOLIS, Program in Genomics, Biodiversity and Land Planning, Campus de Vairão, Universidade do Porto, 4485-661, Vairão Porto Portugal; 9 Sciences Faculty, University of Lisbon, Lisbon, Portugal Sciences Faculty, University of Lisbon Lisbon Portugal; 10 UMR PVBMT- Pôle de Protection des Plantes Université de La Réunion, La Réunion, France UMR PVBMT- Pôle de Protection des Plantes Université de La Réunion La Réunion France

**Keywords:** Azores, Bryoflora, BRYOLAT methodology, elevational gradient, GIMS - Global Island Monitoring Scheme, liverworts, mosses, Pico Island, substrates

## Abstract

**Background:**

In September 2012, a comprehensive survey of Pico Island was conducted along an elevational transect, starting at Manhenha (10 m a.s.l.) and culminating at the Pico Mountain caldera (2200 m a.s.l.). The primary objective was to systematically inventory the bryophytes inhabiting the best-preserved areas of native vegetation environments. Twelve sites were selected, each spaced at 200 m elevation intervals. Within each site, two 10 m x 10 m plots were established in close proximity (10-15 m apart). Within these plots, three 2 m x 2 m quadrats were randomly selected and sampled for bryophytes using microplots measuring 10 cm x 5 cm, which were then collected into paper bags. Six substrates were surveyed in each quadrat: rock, soil, humus, organic matter, tree bark and leaves/fronds. Three replicates were obtained from all substrates available and colonised by bryophytes, resulting in a maximum of 18 microplots per quadrat, 54 microplots per plot, 108 microplots per site, and a total of 1296 microplots across the 12 sites on Pico Island.

**New information:**

Two-thirds of the maximum expected number of microplots (n = 878; 67.75%) were successfully collected, yielding a total of 4896 specimens. The vast majority (n = 4869) were identified at the species/subspecies level. The study identified a total of 70 moss and 71 liverwort species or subspecies. Elevation levels between 600-1000 m a.s.l., particularly in the native forest plots, exhibited both a higher number of microplots and greater species richness. This research significantly enhanced our understanding of Azorean bryophyte diversity and distribution, contributing valuable insights at both local and regional scales. Notably, two new taxa for the Azores were documented during the MOVECLIM study, namely the pleurocarpous mosses *Antitrichiacurtipendula* and *Isotheciuminterludens*.

## Introduction

Bryophytes are ancient terrestrial plants (Laenen et al. 2014), exhibiting different morphological and physiological adaptations that account for their evolutionary success. These plants are characterised by the dominance of the gametophyte over the sporophyte and lack of vascular tissues in the sporophyte. These organisms are taxonomically classified in three Divisions: Bryophyta (mosses), Marchantiophyta (liverworts) and Anthocerotophyta (hornworts) (Goffinet and Shaw 2009). Ecologically, bryophytes occupy a wide range of habitats, from the Poles to the Equator and from sea-level to high mountains, presenting higher diversity in the tropics (Goffinet and Shaw 2009).

As they are small and rootless, bryophytes are very dependent of their immediate context, responding to different environmental changes, namely, substrate composition and acidity, rainfall, temperature, salinity and pollution (e.g. Hodgetts et al. (2019)). Bryophytes maintain a number of ecosystems services, positively influencing the water cycle (Coelho et al. 2023a, Coelho et al. 2023b) and the decomposition of organic material, creating a favourable environment for the development of other non-pioneer organisms (Goffinet and Shaw 2009).

Globally, bryophytes represent the second largest group of plants - with over 20000 species: 13000 mosses, 8000 liverworts and 250 hornworts (Goffinet and Shaw 2009). In Europe, there are 1892 species of bryophytes (1390 mosses; 494 liverworts; 8 hornworts) (Hodgetts et al. 2020), while about a quarter of those are found in the Azores (308 mosses; 162 liverworts; 5 hornworts) (Gabriel et al. 2010), although this archipelago represents only 0.06% of the European territory (Borges et al. 2023). Bryophytes are, thus, one of the main biological groups for maintaining biodiversity (Sérgio et al. 2012), with different taxonomic groups responding differently to environmental pressure and gradients (e.g. Henriques et al. (2016)) and including a number of conservation concern species (Hodgetts et al. 2019).

The distribution of bryophyte species in the nine Azorean islands is uneven, but this fact is not completely explained by area or elevation. For instance, Pico Island, the highest and second largest (2350 m a.s.l.; 445 km^2^), has similar richness of bryophytes (283 species) to the much smaller Faial Island (1043 m a.s.l.; 173 km^2^) (286 species) (Forjaz 2004, Gabriel et al. 2010). This may be related to the sampling effort amongst different islands. Indeed, the work of many collectors and authors contributed to the current knowledge of the distribution of Azorean bryophytes. A list of publications mentioning bryophytes collected in Pico Island may be seen in Suppl. material 1. It comprises 96 references, published from 1862 to 2023, including mostly journal articles (65), but also other types of references, such as books and book chapters, theses and dissertations, scientific reports, Herbaria records and expert's documents. Erik Sjögren stands out as the major source of distribution information, with more than 3000 records on Pico Island; however, the publications of Herman Persson, Jan-Peter Frahm, Pierre and Valentine Allorge, Tutin and Warburg and Juana González-Mancebo, are also noteworthy, having added more than 100 records each to the bryoflora distribution of the Island.

The 292 bryophyte species known to Pico Island (162 mosses; 126 liverworts; 4 hornworts) (Gabriel et al. 2010, Ellis et al. 2016, Hanusch et al. 2020, Sérgio et al. 2022, Sérgio 2023, Sérgio in press), represent ca. 60% of the richness found in the Azores (Borges et al. 2010) and include five Azorean endemics, such as the endangered moss, *Echinodiumrenauldii* (Cardot) Broth. (Coelho et al. 2014b, Gabriel et al. 2018). The composition of the bryoflora in Pico Island is related to the altitudinal gradient, with species richness and abundance responding to elevation, rainfall and substrate diversity (Coelho et al. 2021).

Since 2012, an effort has been made to update the information regarding bryophytes in the Azores, namely under the 'MOVECLIM project – Montane Vegetation as Listening Posts for Climate Change’ (Coelho et al. 2014a, Gabriel et al. 2014, Borges et al. 2018). One of the purposes of this project was to characterise the poorly known, but highly diverse, bryophyte flora of different archipelagos, including the Azores, the Canary Islands and the Mascarene Islands. In the Azores, it was possible to survey seven islands using an adaptation of the BRYOLAT protocol (Gabriel et al. 2014): Pico and Terceira Islands were surveyed in 2012; Flores and São Miguel Islands in 2013; São Jorge and Faial Islands in 2014 and Santa Maria Island in 2019. Graciosa and Corvo Islands, the smallest and devoid of native forest, were not surveyed under this protocol.

Thus, the objective of this study was to inventory bryophyte species in different altitudinal gradients (between 10 m and 2200 m a.s.l.) and substrates (rupicolous, terricolous, humicolous, lignicolous, epiphytic, epiphyllous) in the Island of Pico sampled in a stratified way.

## General description

### Purpose

The main objective of this work was to inventory the bryoflora present in the Pico Natural Park, including Municipalities of Lajes do Pico, São Roque do Pico and Madalena. Sampling was carried out on different altitudinal levels (between 10 m and 2200 m) and substrates (rupicolous, terricolous, humicolous, lignicolous, epiphytic, epiphyllous), with the aim of expanding the database of bryophyte species (mosses, liverworts, hornworts) known to Pico Island.

## Project description

### Title

Inventory of bryoflora present in different altitudinal gradients of Pico Island (Azores).

### Personnel

The bryoflora inventory of Pico Island was carried out during the month of September 2012, within the dates 5-10, under the responsibility of Rosalina Gabriel (RG) and Claudine Ah-Peng (CAP), with the participation of Márcia C.M. Coelho (MC), Débora Henriques (DH), Silvia Calvo Aranda (SCA) and Fernando Pereira (FP). Species growing on organic matter, tree trunks and leaves were identified by MC supervised by RG, while Helena Hespanhol (HH) identified species growing on rock and soil. Challenging specimens were confirmed by Cecília Sérgio (CS) and Manuela Sim-Sim (MSS). In 2023, the species of the genus *Frullania* were reviewed by Leila N. Morgado (LNM) and RG and were later confirmed by MSS.

### Study area description

**Figure 1.**
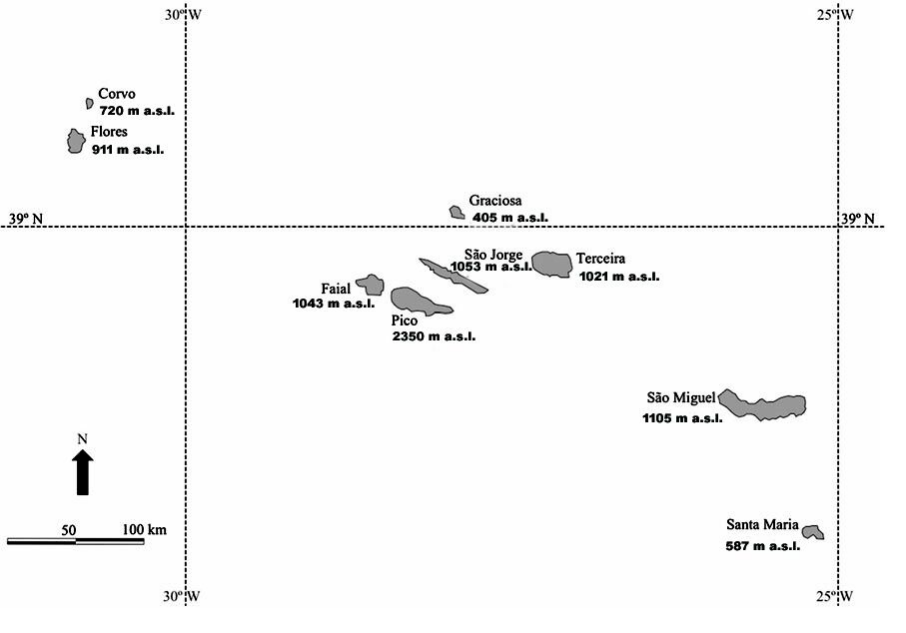
Location and maximum elevation of each island of the Azores Archipelago. Source: Section of Management and Environmental Planning, University of the Azores; Elevation values follow Forjaz (2004).

The Azores Archipelago is a Portuguese autonomous region and comprises nine islands of volcanic origin. It is located in the North Atlantic Ocean (Fig. 1), about 1500 km from the western coast of mainland Europe and approximately 3900 km from the North America coasts. The shortest distance to European Portuguese coast is about 1400 km (Forjaz 2004).

Given its oceanic location, the Azores have a typically maritime climate, which translates into mild tempera­tures, with a small temperature range, high relative humidity (%) and high rainfall (mm) in autumn and winter. The average annual temperature varies between 12°C and 23°C, the average annual rainfall varies between 1000 and 1600 mm at sea level and the relative humidity is usually quite high, largely exceeding 80% in all seasons (Forjaz 2004, IPMA 2022).

The Laurissilva Forest of Macaronesia was recognised as a World Natural Heritage site by UNESCO in 1999 (CONSLEG (1992L0043) 2014). Together with the Juniperus-Ilex montane cloud forests, these formations present the highest number of endemisms and other indigenous species (Nunes et al. 2015, Schäfer 2021, Elias et al. 2022). In fact, there are approximately 452 endemic species amongst land and freshwater organisms in the Azores (Borges et al. 2010) and vascular plants stand out with 73 Azorean endemic species and subspecies, a value surpassed only by phylum Arthropoda (266 taxa) (Borges et al. 2010). Native vegetation also includes a high number and cover of bryophytes (Gabriel and Bates 2005, Homem and Gabriel 2008, Couto 2010, Gabriel et al. 2011, Elias et al. 2022), some of which rare and endangered (Esquivel et al. 2008, Hodgetts et al. 2019).

Pico Island (38º33'57" and 38º33'44" N and 28º01'39" and 28º32'33" W), belongs to the central group of the Azores Archipelago (Portugal) in the North Atlantic Ocean. This Island is the second largest (445 km^2^), the highest (2350 m a.s.l.) and the youngest (0.27 MY) of the Azores (Forjaz 2004) and its forest incorporates the highest contribution of endemic (44%) and native (38%) taxa, compared to the Islands of São Miguel and Terceira (Borges Silva et al. 2022).

**Figure 2.**
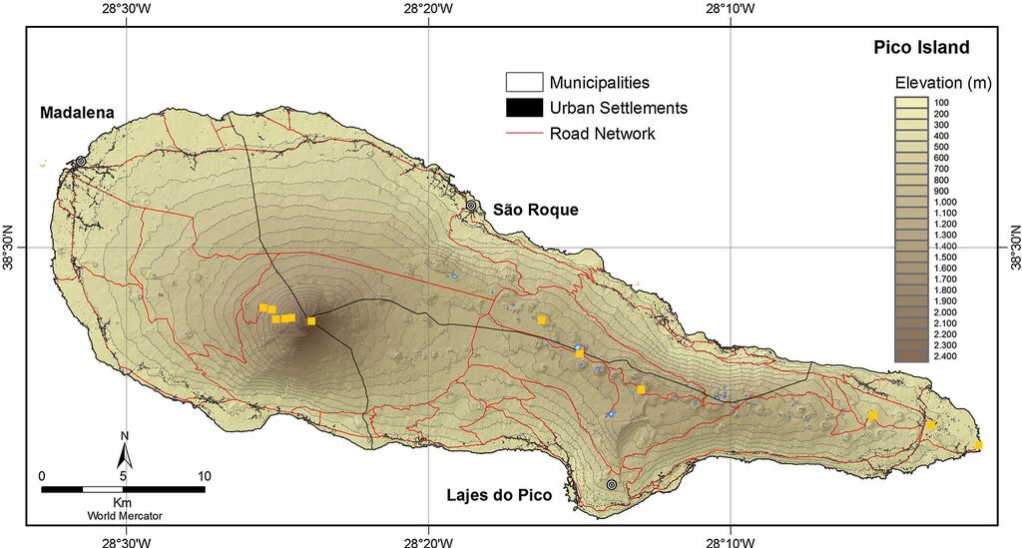
Map of Pico Island with indications of sampling points (yellow squares) (Source: Gil et al. 2017, with modifications).

The sampling location and coordinates are listed in Table 1 and Fig. 2.

### Design description

The sampling was performed in 2012, during 5-10 September, along a longitudinal elevational transect (east to west) in the Pico Natural Park, including the best-preserved areas of native vegetation. The samples were collected in different altitudinal gradients (10 - 2200 m) and substrates (rupicolous, terricolous, humicolous, lignicolous, epiphytic, epiphyllous).

### Funding

This study was financed by ERANET BIOME MOVECLIM – ‘Montane vegetation as listening posts for climate change’ of the regional government of the Azores, grant number M2.1.2/F/04/2011/NET.

MCMC was funded by the FUNDO REGIONAL PARA A CIÊNCIA E TECNOLOGIA (FRCT) of the Regional Government of the Azores, grant number M3.1.2/F/007/2012.

RG, LN and PAVB are currently funded by FCT-UIDB/00329/2020-2024 (Thematic Line 1–integrated ecological assessment of environmental change on biodiversity) and Azores DRCT Pluriannual Funding (M1.1.A/FUNC.UI&D/010/2021-2024).

## Sampling methods

### Study extent

Pico Island has the highest plant diversity compared to the other islands in the Archipelago, mainly due to a higher number of altitudinal vegetation areas (Dias et al. 2005). The soils are relatively young, mostly composed of basaltic rock debris (i.e. leptosols), presenting lower values of carbon accumulation (Borges Silva et al. 2022). This study was carried out in the Pico National Park (Azores), which has a large protected area (156 km^2^ - terrestrial, 79 km^2^ - maritime), including the mountain (the central volcanic formation that reaches 2351 m elevation), native forests and woodlands, lagoons and the coastal zones.

### Sampling description

**Figure 3.**
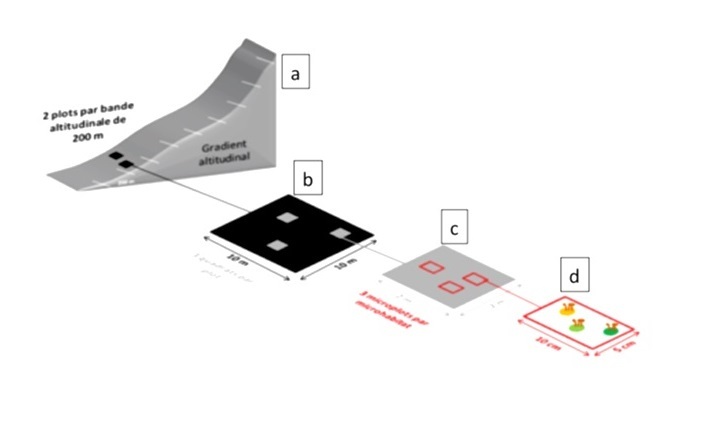
Altitudinal sampling model on Pico Island (Ah-Peng et al. 2012 and Gabriel et al. 2014): (a) 200 m elevation steps, two plots (black squares, 10 m x 10 m) are placed within 10 m to 15 m from each other; (b) each plot is divided into 25 quadrats (grey squares, 2 m x 2 m), from which three are sampled; (c) and (d) – each quadrat is thoroughly examined for different substrata and three microplots (red rectangular shapes, 5 cm x 10 cm) are collected on every microhabitat, except on trees, where nine replicates are made, at three different height levels. (Source: Gabriel et al. (2014)).

The field study was carried out according to the BRYOLAT methodology (Ah-Peng et al. 2012), with some modifications according to the conditions and knowledge of the Azores flora (Gabriel et al. 2014). In each plot, three quadrats (2 m x 2 m) were randomly selected and bryophytes were sampled in a total of three microplots (5 cm x 10 cm) in each different substrate (rupicolous, terricolous, humicolous, lignicolous, epiphytic, epiphyllous) and altitudinal gradients (between 10 m and 2200 m). This methodology consists of sampling the bryoflora according to the diagram (Fig. 3, Gabriel et al. (2014)). In the laboratory, the abundance and sociability of the bryophyte species were identified and estimated.

### Quality control

FIELD: An initial visit of Pico Island was made by PAVB and RG in July 2012 to prospect the most suitable sampling sites. Plots were placed within homogeneous areas of representative native vegetation found at each sampled elevation. Sampling was made by experienced bryologists, who ensured the samples were properly collected, while avoiding the excessive removal of material. STORAGE: After the collection of the microplots in paper bags, these were left open and separated in a darkened room until complete dehydration. After identification, every sample was transferred to herbarium envelopes properly identified. All these envelopes were stored in the Herbarium of the University of the Azores (AZU), Section Bryophytes, under the name “MOVECLIM – AZORES project: Bryophytes from Pico Island (2012)”. TAXONOMY: All efforts were made to achieve an accurate identification of the specimens: (i) the most updated keys and floras were used by/under the supervision of experienced bryologists; (ii) challenging samples were sent to specialists for confirmation/identification; (iii) identification of extremely small or etiolated specimens was not pursued to the species level. Mosses were identified mainly using the floras of Smith (2004) and Casas et al. (2006), whereas liverworts were identified mainly using the floras written by Paton (1999) and Casas et al. (2009) and the taxonomic key of Schumacker and Vána (2005). Visual guides (e.g. Atherton et al. (2010), Lüth (2019)) were also consulted, as well as the BBS Field Guide online pages, the Bildatlas der Moose Deutschlands for morphological and ecological data. Nomenclature follows Gabriel et al. (2010) and updates available on the Azorean Biodiversity Portal (Hodgetts et al. 2020, Borges et al. 2023). Species identification was performed by Márcia Catarina Mendes Coelho, under the supervision of Rosalina Gabriel and by Helena Hespanhol. In 2023, all the *Frullania* specimens were reviewed by Leila Nunes Morgado under the supervision of Rosalina Gabriel. The identification of some challenging specimens was performed by Manuela Sim-Sim and Cecília Sérgio. REPRESENTATION OF THE PICO BRYOFLORA: Species accumulation curves were generated, based on a presence–absence microplot-scale matrix using Chao 2 estimator. Sampling completeness was high both for liverworts (87.5%) and mosses (94.5%) (Coelho et al. 2021).

## Geographic coverage

### Description

The study was carried out in Pico Island (Azores Archipelago, Portugal). The 12 sampling sites were distributed amongst the three municipalities of the island: Lajes do Pico, São Roque do Pico and Madalena; the coordinates range between the following values:

### Coordinates

38.41375 and 38.47064 Latitude; -28.0298 and -28.42525 Longitude.

## Taxonomic coverage

### Description

Bryophytes, including specimen from Division Bryophyta (mosses) and Division Marchantiophyta (liverworts). No elements from Division Anthocerotophyta were collected during this survey.

## Temporal coverage

### Notes

The sampling was performed in 2012, 5-10 September.

## Usage licence

### Usage licence

Creative Commons Public Domain Waiver (CC-Zero)

### IP rights notes

Additional information on this study may also be requested from the corresponding author.

## Data resources

### Data package title

The MOVECLIM – AZORES project: Bryophytes from Pico Island (2012).

### Resource link

https://www.gbif.org/dataset/88d3beab-eb7c-4a3a-927e-7b8bf6d35ef6

### Alternative identifiers

http://ipt.gbif.pt/ipt/resource?r=bryophytes_pico_2012

### Number of data sets

2

### Data set 1.

#### Data set name

Event table

#### Data format

Darwin Core Archive

#### Character set

UTF-8

#### Download URL

http://ipt.gbif.pt/ipt/resource?r=bryophytes_pico_2012

#### Data format version

1.4

#### Description

The dataset was published in the Global Biodiversity Information Facility platform, GBIF (Gabriel et al. 2023). The following data table includes all the records for which a taxonomic identification of the species was possible. The dataset submitted to GBIF is structured as a sample event dataset that has been published as a Darwin Core Archive (DwCA), which is a standardised format for sharing biodiversity data as a set of one or more data tables. The core data file contains 878 records (eventID). This GBIF IPT (Integrated Publishing Toolkit, Version 2.5.6) archives the data and, thus, serves as the data repository. The data and resource metadata are available for download in the Portuguese GBIF Portal IPT (Gabriel et al. 2023).

**Data set 1.** 

Column label	Column description
id	Identifier of the events, unique for the dataset.
type	Type of the record, as defined by the Dublin Core Standard.
datasetName	Name of the dataset that in current projects is "MOVECLIM-AZO-PIC_2012_Bryophytes from Pico Island".
eventID	Identifier of the events, unique for the dataset.
samplingProtocol	The sampling protocol used to capture the species. Detailed description of the sampling methodology in the field. Two plots of 10 m × 10 m (P1, P2), were set out at the best-preserved areas of native vegetation sites, every 200 m elevation, across an elevation gradient from coastal areas to Pico summit. Each plot was subdivided into 25 quadrats (2 m × 2 m), from which three were randomly selected for the collection of bryophyte species. Within each quadrat, bryophytes were collected in small sampling units (microplots of 10 cm × 5 cm), obtaining, whenever possible, three replicates per surveyed substrate (RU, rock, TE, soil, HU, humus, LI, dead wood, T, bark at three heights from the tree [a, 1-50 cm; b, 51-100 cm; c, 101-200 cm], LF, leaves/fronds).
minimumElevationInMetres	The lower limit of the range of elevation (altitude above sea level) of the Location.
eventDate	The date-time or interval during which an Event occurred. For occurrences, this is the date-time when the event was recorded.
year	Year the sample was collected (2012).
habitat	The habitat for an Event.
continent	The name of the continent in which the Location occurs (Europe).
islandGroup	The name of the island group in which the Location occurs (Azores).
island	The name of the island on or near which the Location occurs (Pico Island).
country	The name of the country or major administrative unit in which the Location occurs (Portugal).
countryCode	The standard code for the country in which the Location occurs (PT).
municipality	The full, unabbreviated name of the next smaller administrative region than county (city, municipality etc.) in which the Location occurs.
locality	The specific description of the place.
verbatimCoordinates	Original coordinates recorded.
decimalLatitude	Approximate centre point decimal latitude of the field site in GPS coordinates.
decimalLongitude	Approximate centre point decimal longitude of the field site in GPS coordinates.
geodeticDatum	Standard Global Positioning System coordinate reference for the location of the sample collection points.
coordinateUncertaintyInMetres	Uncertain value of coordinate metrics.
coordinatePrecision	Value in decimal degrees to a precision of five decimal places.
georeferenceSources	Navigation system used to record the location of sample collections.

### Data set 2.

#### Data set name

Occurrence Table

#### Data format

Darwin Core Archive

#### Character set

UTF-8

#### Download URL

http://ipt.gbif.pt/ipt/resource?r=bryophytes_pico_2012

#### Data format version

1.4

#### Description

The dataset was published in the Global Biodiversity Information Facility platform, GBIF (Gabriel et al. 2023). The following data table includes all the records for which a taxonomic identification of the species was possible. The dataset submitted to GBIF is structured as an occurrence table that has been published as a Darwin Core Archive (DwCA), which is a standardised format for sharing biodiversity data as a set of one or more data tables. The core data file contains 4896 records (occurrenceID). This GBIF IPT (Integrated Publishing Toolkit, Version 2.5.6) archives the data and, thus, serves as the data repository. The data and resource metadata are available for download in the Portuguese GBIF Portal IPT (Gabriel et al. 2023).

**Data set 2.** 

Column label	Column description
eventID	Identifier of the events, unique for the dataset.
licence	Reference to the licence under which the record is published.
institutionID	The identity of the institution publishing the data.
institutionCode	The code of the institution publishing the data.
collectionCode	The code of the collection where the specimens are conserved.
datasetName	Project reference.
type	Characteristics of the object of study.
basisOfRecord	The nature of the data record.
dynamicProperties	A list of additional measurements, facts, characteristics or assertions about the record, including IUCN categories (Endangered, Vulnerable, Near Threatened, Least Concern, Not Evaluated) and colonisation status of taxa following the standard notation used for bryophytes (Azorean endemic, Macaronesian endemic, Ibero-Macaronesian endemic, European endemic, non-endemic).
occurrenceID	Identifier of the record, coded as a global unique identifier.
recordNumber	An identifier given to the Occurrence at the time it was recorded.
recordedBy	A list (concatenated and separated) of names of people, groups or organisations responsible for recording the original Occurrence.
identifiedBy	A list (concatenated and separated) of names of people, who made the identification.
disposition	The current state of a specimen with respect to the collection identified in collectionCode or collectionID.
taxonRank	Lowest taxonomic rank of the record.
kingdom	Kingdom name.
phylum	Phylum name.
class	Class name.
order	Order name.
family	Family name.
genus	Genus name.
specificEpithet	Specific epithet.
infraspecificEpithet	Infraspecific epithet at subspecies level.
scientificNameAuthorship	The authorship information for the scientificName formatted according to the conventions of the applicable nomenclaturalCode.
scientificName	Complete scientific name including author.
organismQuantity	A number or enumeration value for the quantity of organisms (i, solitary specimen - one or few individuals; p, occasional and less than 5% cover; 1, less than 5% cover of total area; 2, 5%-25% of total area; 3, 25%-50% of total area; 4, 50%-75% of total area; 5, 75%-100% of total area).
organismQuantityType	Braun-Blanquet Scale.
establishmentMeans	The process of establishment of the species in the location, using a controlled vocabulary: 'native non-endemic', 'introduced', 'endemic'.
occurrenceRemarks	Remarks on the occurrence substrate from where the specimens were captured.

## Additional information

**Figure 4.**
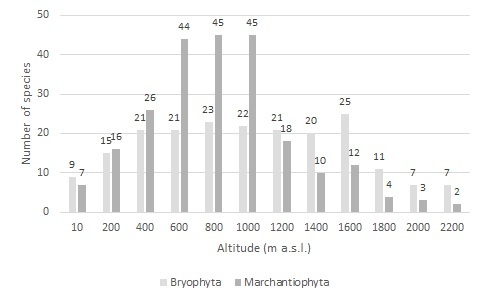
The richness of species of mosses (Division Bryophyta) and liverworts (Division Marchantiophyta) along Pico Island’s elevational gradient.

**Figure 5.**
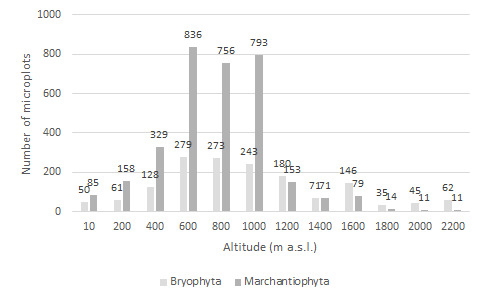
The number of presences in 50 cm^2^ microplots of species of mosses (Division Bryophyta) and liverworts (Division Marchantiophyta) along Pico Island’s elevational gradient.

The 878 events yielded a grand total of 4896 specimens, with the majority (n = 4869; 99.45%) being successfully identified down to the species/subspecies level. Division Bryophyta is represented by 70 species, belonging to three classes (Bryopsida, Polytrichopsida and Sphagnopsida), 10 orders, 28 families and 45 genera, while Division Marchantiophyta is represented by 71 species, including three subspecies, organised in two classes (Jungermanniopsida and Marchantiopsida), six orders, 24 families and 43 genera (see Occurrence Table at Gabriel et al. (2023)). Both the number of species (Fig. 4) and the number of records (Fig. 5) show a peak from 600 m to 1000 m altitude, which correspond to the sites with more complex vegetation (Coelho et al. 2014a, Coelho et al. 2014b, Coelho et al. 2021).

Considering the colonisation status, all the taxa can be considered native, but four are Azoren endemics, the liverworts *Bazzaniaazorica* H.Buch & Perss. and Leptoscyphusporphyriussubsp.azoricus (H.Buch & Perss.) Vanderp. & Heirichs and the pleurocarpic mosses *Echinodiumrenauldii* (Cardot) Broth. and *Rhynchostegiellaazorica* Hedenäs & Vanderp.; besides, a total of nine species are Macaronesian endemics, three are Iberian-Macaronesian endemics and seven are European endemics (see Table 2).

Almost two-thirds of the bryophytes were collected in three of the 12 elevation levels: at 600 m a.s.l. (22.90%), 800 m (21.13%) and 1000 m (21.28%); that proportion is much higher for liverworts than mosses (72.36% vs. 50.54%) (Figs 4, 5).

## Supplementary Material

3BCA68F6-CD3B-52E8-9EF1-3B9EFC2250FD 10.3897/BDJ.12.e117890.suppl1 Supplementary material 1 List of publications mentioning bryophytes in Pico Island (Azores) - 1862-2023 Data type Table Brief description List of references mentioning the distribution of bryophytes - mosses, liverworts and hornworts - in Pico Island (Azores, Portugal). Each reference includes also information on the year of publication and type. File: oo_958534.txt https://binary.pensoft.net/file/958534 Rosalina Gabriel

## Figures and Tables

**Table 1.** Bryophyte sampling plots in the different altitudinal gradients (10-2200 m), location name, elevation (in metres) and coordinates (in decimals) (Pico Island, Azores).

**Plot code**	**Locality**	**Elevation (m a.s.l.)**	**Latitude**	**Longitude**
PIC_0010_P1	Pico, Lajes do Pico, 0010 m, Manhenha - Farol.	17	38.413750	-28.029806
PIC_0010_P2		14	38.413750	-28.029944
PIC_0200_P1	Pico, Lajes do Pico, 0200 m, Cabeço da Hera.	224	38.418190	-28.053940
PIC_0200_P2		226	38.418167	-28.053639
PIC_0400_P1	Pico, Lajes do Pico, 0400 m, Piedade - Fetais.	365	38.425778	-28.087639
PIC_0400_P2		364	38.425917	-28.087361
PIC_0600_P1	Pico, São Roque do Pico, 0600 m, Caminho dos Burros - Chão Verde.	621	38.468847	-28.276194
PIC_0600_P2		623	38.468814	-28.275944
PIC_0800_P1	Pico, São Roque do Pico, 0800 m, Caiado.	809	38.455778	-28.257278
PIC_0800_P2		813	38.455667	-28.257250
PIC_1000_P1	Pico, Lajes do Pico, 1000 m, Caveiro.	952	38.437167	-28.213028
PIC_1000_P2		947	38.437167	-28.212806
PIC_1200_P1	Pico, Madalena, 1200 m, Trilho da Montanha.	1261	38.470361	-28.425250
PIC_1200_P2		1261	38.470639	-28.425028
PIC_1400_P1	Pico, Madalena, 1400 m, Trilho da Montanha.	1418	38.469583	-28.421361
PIC_1400_P2		1407	38.469306	-28.421250
PIC_1600_P1	Pico, Madalena, 1600 m, Trilho da Montanha.	1601	38.465972	-28.416528
PIC_1600_P2		1588	38.465472	-28.416583
PIC_1800_P1	Pico, Madalena, 1800 m, Trilho da Montanha.	1805	38.466083	-28.412556
PIC_1800_P2		1802	38.465944	-28.412500
PIC_2000_P1	Pico, Madalena, 2000 m, Trilho da Montanha.	2003	38.465722	-28.408333
PIC_2000_P2		2009	38.465583	-28.408111
PIC_2200_P1	Pico, Madalena, 2200 m, Trilho da Montanha, Cratera antes do Piquinho.	2239	38.466361	-28.399222
PIC_2200_P2		2242	38.466722	-28.399417

**Table 2.** List of sampled species and subspecies in each of the colonisation status categories (AZ, Azorean; MAC, Macaronesian; IB-MAC, Ibero-Macaronesian; EUR, European).

**Colonization status**	**Scientific Name**
**Division: Bryophyta**	
Azorean endemic	*Echinodiumrenauldii* (Cardot) Broth.
	*Rhynchostegiellaazorica* Hedenäs & Vanderp.
Macaronesian endemic	*Alophosiaazorica* (Renauld & Cardot) Cardot
	*Andoaberthelotiana* (Mont.) Ochyra
	*Isotheciumprolixum* (Mitt.) M.Stech, Sim-Sim, Tangney & D.Quandt
	*Thamnobryummaderense* (Kindb.) Hedenäs
Ibero-Macaronesian endemic	*Tetrastichiumfontanum* (Mitt.) Cardot
	*Tetrastichiumvirens* (Cardot) S.P.Churchill
European endemic	*Hypnumuncinulatum* Jur.
	*Pseudotaxiphyllumlaetevirens* (Dixon & Luisier ex F.Koppe & Düll) Hedenäs
	*Ulotacalvescens* Wilson
Native	*Antitrichiacurtipendula* (Hedw.) Brid.
	*Brachytheciastrumvelutinum* (Hedw.) Ignatov & Huttunen
	*Brachytheciummildeanum* (Schimp.) Schimp.
	*Campylopusflexuosus* (Hedw.) Brid.
	*Campylopusfragilis* (Brid.) Bruch & Schimp.
	*Campylopuspilifer* Brid.
	*Campylopuspyriformis* (Schultz) Brid.
	*Campylopusshawii* Wilson
	*Cyclodictyonlaetevirens* (Hook. & Taylor) Mitt.
	*Daltonialindigiana* Hampe
	*Dicranellaheteromalla* (Hedw.) Schimp.
	*Dicranumflagellare* Hedw.
	*Dicranumscottianum* Turner
	*Diphysciumfoliosum* (Hedw.) D.Mohr
	*Ditrichumsubulatum* Hampe
	*Fissidensbryoides* Hedw.
	*Fissidensdubius* P.Beauv.
	*Fissidensserrulatus* Brid.
	*Fissidenstaxifolius* Hedw.
	*Fissidensviridulus* (Sw.) Wahlenb.
	*Grimmiaelongata* Kaulf.
	*Heterocladiumheteropterum* (Brid.) Schimp.
	*Heterocladiumwulfsbergii* I.Hagen
	*Hylocomiumsplendens* (Hedw.) Schimp.
	*Hymenolomacrispulum* (Hedw.) Ochyra
	*Hypnumcupressiforme* Hedw.
	*Isotheciuminterludens* Stirt.
	*Isotheciummyosuroides* Brid.
	*Kiaeriablyttii* (Bruch & Schimp.) Broth.
	*Leucobryumglaucum* (Hedw.) Ångstr.
	*Leucobryumjuniperoideum* (Brid.) Müll.Hal.
	*Mniumhornum* Hedw.
	*Myuriumhochstetteri* (Schimp.) Kindb.
	*Plagiomniumundulatum* (Hedw.) T.J.Kop.
	*Polytrichumcommune* Hedw.
	*Polytrichumformosum* Hedw.
	*Polytrichumjuniperinum* Hedw.
	*Polytrichumpiliferum* Hedw.
	*Pseudorhynchostegielladuriaei* (Mont.) Ignatov & Vanderp.
	*Pseudoscleropodiumpurum* (Hedw.) M.Fleisch.
	*Ptychostomumtorquescens* (Bruch & Schimp.) Ros & Mazimpaka
	*Racomitriumaffine* (F.Weber & D.Mohr) Lindb.
	*Racomitriumfasciculare* (Hedw.) Brid.
	*Racomitriumheterostichum* (Hedw.) Brid.
	*Racomitriumlanuginosum* (Hedw.) Brid.
	*Rhynchostegiumconfertum* (Dicks.) Schimp.
	*Rhytidiadelphusloreus* (Hedw.) Warnst.
	*Rhytidiadelphussquarrosus* (Hedw.) Warnst.
	*Sciuro-hypnum plumosum* (Hedw.) Ignatov & Huttunen
	*Sematophyllumsubstrumulosum* (Hampe) E.Britton
	*Serpoleskeaconfervoides* (Brid.) Schimp.
	*Sphagnumpalustre* L.
	*Thamnobryumalopecurum* (Hedw.) Gangulee
	*Thuidiumtamariscinum* (Hedw.) Schimp.
	*Tortellaflavovirens* (Bruch) Broth.
	*Trichostomumbrachydontium* Bruch
	*Ulotacrispa* (Hedw.) Brid.
	*Zygodonconoideus* (Dicks.) Hook. & Taylor
	*Zygodonviridissimus* (Dicks.) Brid.
**Division: Marchantiophyta**	
Azorean endemic	*Bazzaniaazorica* H.Buch & Perss.
	Leptoscyphusporphyriussubsp.azoricus (H.Buch & Perss.) Vanderp. & Heirichs
Macaronesian endemic	*Calypogeiaazorica* Bischl.
	*Cheilolejeuneacedercreutzii* (H.Buch & Perss.) Grolle
	*Heteroscyphusdenticulatus* (Mitt.) Schiffn.
	*Radulawichurae* Steph.
	*Telaraneaazorica* (H.Buch & Perss.) Pócs
Ibero-Macaronesian endemic	*Frullaniaazorica* Sim-Sim, Sérgio, Mues & Kraut
European endemic	*Frullaniamicrophylla* (Gottsche) Pearson
	*Lejeuneahibernica* Bischl., H.A.Mill. & Bonner ex Grolle
	*Radulaholtii* Spruce
	*Saccogynaviticulosa* (L.) Dumort.
Native	*Acrobolbusazoricus* (Grolle & Perss.) Briscoe
	*Aneurapinguis* (L.) Dumort.
	*Blepharostomatrichophyllum* (L.) Dumort.
	*Calypogeiaarguta* Nees & Mont.
	*Calypogeiafissa* (L.) Raddi
	*Calypogeiamuelleriana* (Schiffn.) Müll.Frib.
	*Calypogeiasphagnicola* (Arnell & J.Perss.) Warnst. & Loeske
	*Cephaloziabicuspidata* (L.) Dumort.
	*Cololejeuneaazorica* V.Allorge & Jovet-Ast
	*Cololejeuneamicroscopica* (Taylor) Schiffn.
	*Cololejeuneasintenisii* (Steph.) Pócs
	*Coluracalyptrifolia* (Hook.) Dumort.
	*Diplophyllumalbicans* (L.) Dumort.
	*Drepanolejeuneahamatifolia* (Hook.) Schiffn.
	*Frullaniaacicularis* Hentschel & von Konrat
	*Frullaniateneriffae* (F.Weber) Nees
	*Fuscocephaloziopsiscrassifolia* (Lindenb. & Gottsche) Váňa & L.Söderstr.
	*Geocalyxgraveolens* (Schrad.) Nees
	*Gymnomitrionadustum* Nees
	*Harpalejeuneamolleri* (Steph.) Grolle
	*Herbertusazoricus* (Steph.) P.W.Richards
	*Herbertusborealis* Crundw.
	*Lejeuneaeckloniana* Lindenb.
	Lejeuneaflavasubsp.moorei (Lindb.) R.M.Schust.
	*Lejeunealamacerina* (Steph.) Schiffn.
	*Lejeuneapatens* Lindb.
	*Lepidoziacupressina* (Sw.) Lindenb. subsp. cupressina
	*Lepidoziareptans* (L.) Dumort.
	*Lophocoleacoadunata* (Sw.) Mont.
	*Lophocoleafragrans* (Moris & De Not.) Gottsche, Lindenb. & Nees
	*Lophocoleaheterophylla* (Schrad.) Dumort.
	*Marchantiapolymorpha* L.
	*Marchesiniamackaii* (Hook.) Gray
	*Marsupellasparsifolia* (Lindb.) Dumort.
	*Metzgeriafurcata* (L.) Corda
	*Mniolomafuscum* (Lehm.) R.M.Schust.
	*Myriocoleopsisminutissima* (Sm.) R.L.Zhu, Y.Yu & Pócs
	*Nardiascalaris* Gray
	*Nowelliacurvifolia* (Dicks.) Mitt.
	*Odontoschismadenudatum* (Mart.) Dumort.
	*Pallavicinialyellii* (Hook.) Gray
	*Pelliaepiphylla* (L.) Corda
	*Plagiochilabifaria* (Sw.) Lindenb.
	*Plagiochilaexigua* (Taylor) Taylor
	*Plagiochilapunctata* (Taylor) Taylor
	*Plagiochilaretrorsa* Gottsche
	*Porellacanariensis* (F.Weber) Underw.
	*Porellaobtusata* (Taylor) Trevis.
	*Pseudomarsupidiumdecipiens* (Hook.) Grolle
	*Radulaaquilegia* (Hook.f. & Taylor) Gottsche, Lindenb. & Nees
	*Radulacarringtonii* J.B.Jack
	*Radulacomplanata* (L.) Dumort.
	*Rebouliahemisphaerica* (L.) Raddi
	*Riccardiachamedryfolia* (With.) Grolle
	*Riccardiamultifida* (L.) Gray
	*Scapaniagracilis* Lindb.
	*Scapaniascandica* (Arnell & H.Buch) Macvicar
	*Telaraneaeuropaea* J.J.Engel & G.L.Merr.
	Jubulahutchinsiae(Hook.)Dumort.subsp.hutchinsiae
